# A comparison of video laryngoscopy and direct laryngoscopy in critically ill patients

**DOI:** 10.1186/s13054-024-04811-8

**Published:** 2024-01-21

**Authors:** Yang Zhao, Qian Wang, Bin Zang

**Affiliations:** 1 Department of Critical Care Medicine, Shengjing Hospital of China Medical University, Shenyang, 110000 China; 2 Department of Emergency, The Fourth Affiliated Hospital of China Medical University, Shenyang, 110000 China

## To the Editor,

We read the article “Video versus direct laryngoscopy in critically ill patients: an updated systematic review and meta-analysis of randomized controlled trials” by Araújo et al. [1] with great interest. Although the article is well-written, certain parts merit further discussion.

**Fig. 1:**
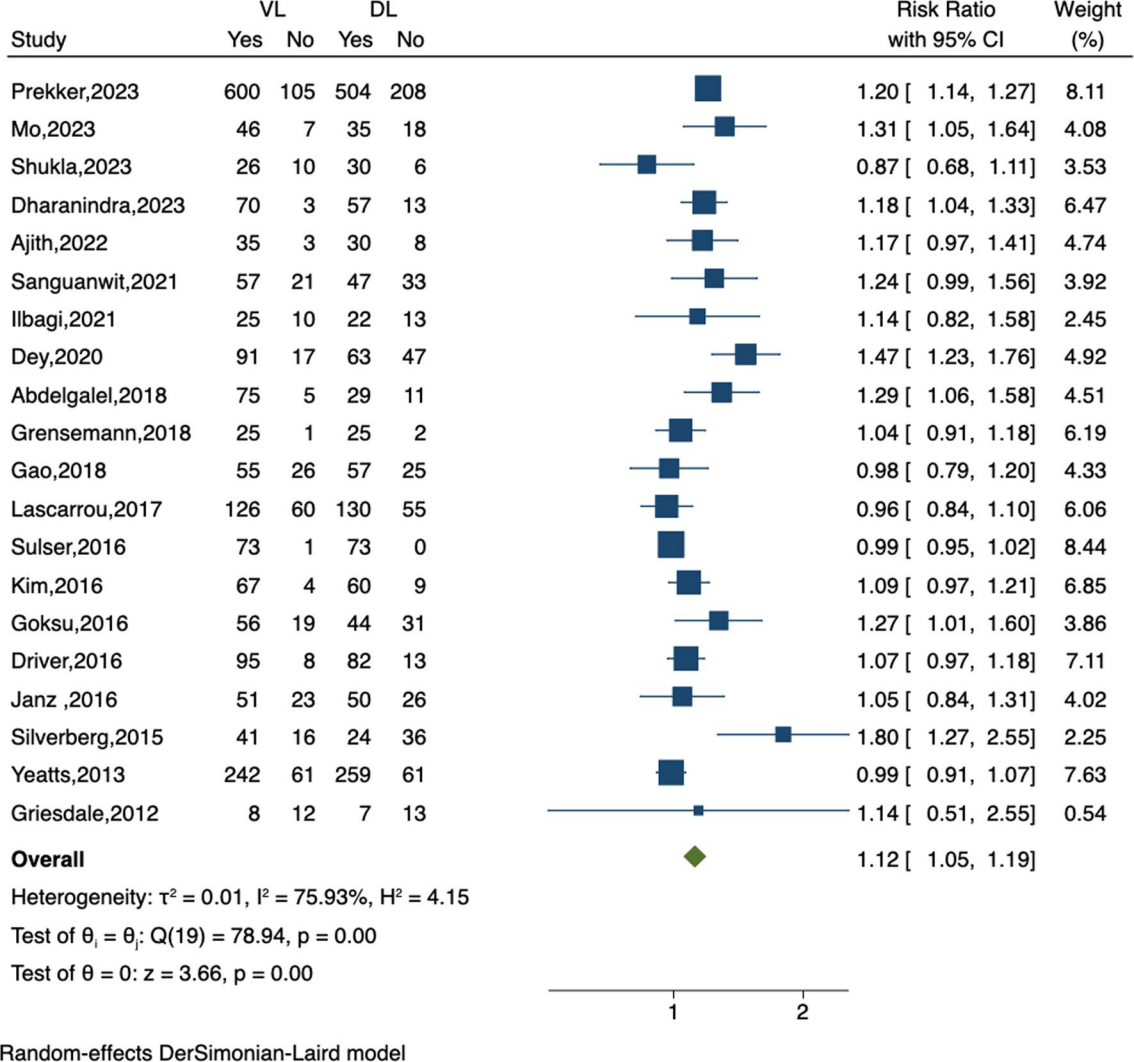
Forest plot of the first-attempt success rate in endotracheal intubation using video laryngoscopy compared to direct laryngoscopy. VL, video laryngoscopy; DL, direct laryngoscopy; CI, confidence interval

Following the authors' search strategy, our reevaluation revealed that the study omitted several randomized controlled trials (RCTs) that met the criteria, including Mo et al. [2], Shukla et al. [3], Ilbagi et al. [4], Grensemann et al. [5], Kim et al. [6], Silverberg et al. [7]. These six additional RCTs increased the total patient count to 4532, with 2276 in the video laryngoscopy (VL) group and 2256 in the direct laryngoscopy (DL) group. We extracted data from the newly included RCTs and analyzed successful intubations on the first attempt using STATA 16.0 (Stata Corp., College Station, TX, USA). The meta-analysis result suggests that VL significantly enhances the first-attempt success rate compared to DL (RR, 1.12; 95% CI 1.05, 1.19;* P* < 0.05) (Fig. 1).

**Fig. 2:**
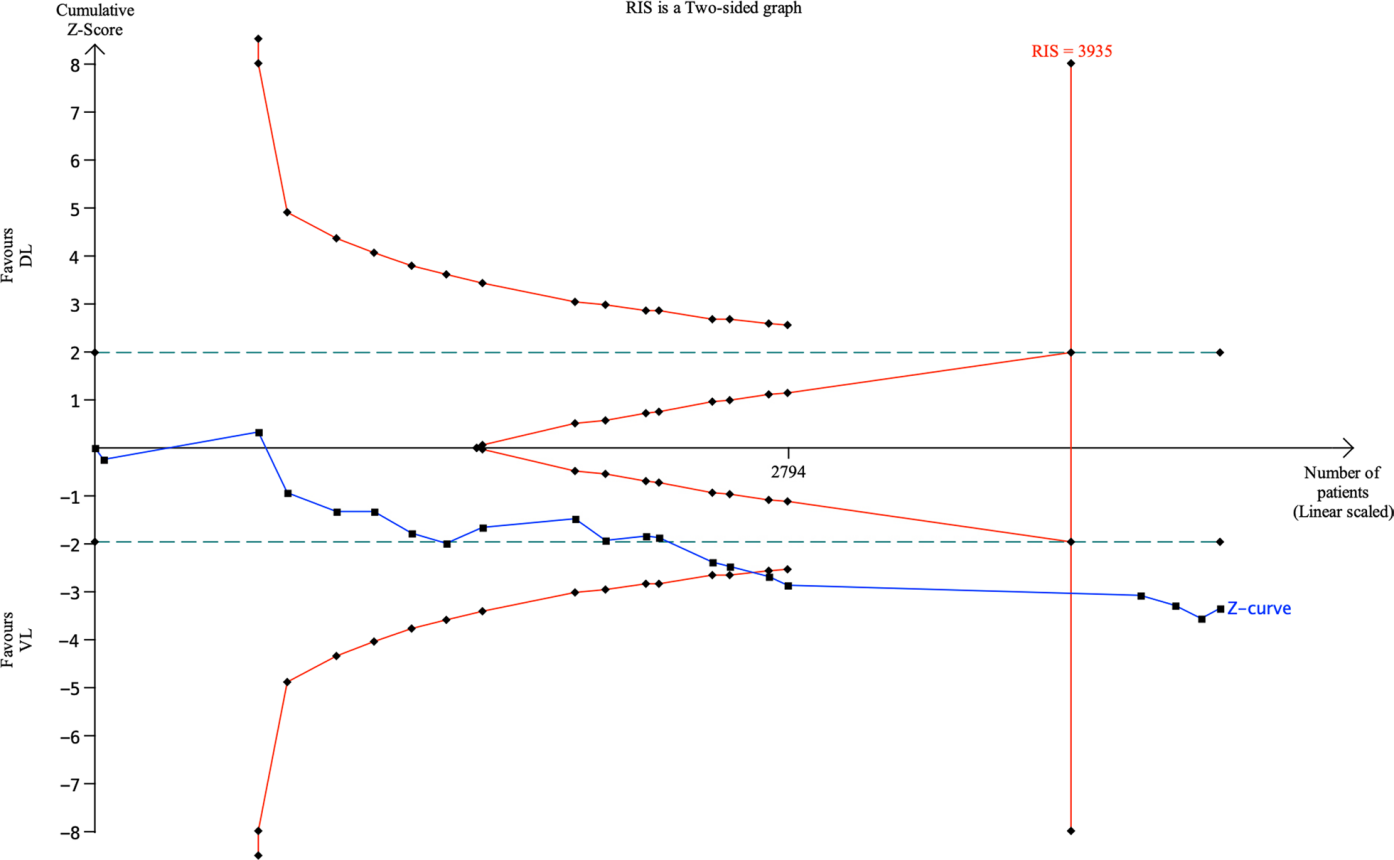
Trial sequential analysis (TSA) of the first-attempt success rate. The blue Z curve represents the treatment effect (pooled relative risk). Green dotted lines denote traditional boundaries, and red solid lines indicate TSA boundaries. RIS, required information size; VL, video laryngoscopy; DL, direct laryngoscopy

In light of the new result regarding the first-attempt success rate, we conducted a Trial Sequential Analysis (TSA) analysis. The two-sided Type I error was set at 5%, and a power of 80% was chosen to calculate the required information size (RIS) for the analysis. The incidence in the control arm was estimated through the meta-analysis. The results showed that the blue cumulative Z-curve, created using a random-effects model, crossed the traditional and TSA boundaries and reached the RIS. Consequently, this finding confirmed the improved first-attempt success rate with VL (Fig. 2).

By expanding the sample size and increasing the number of studies, our analysis provided more comprehensive evidence-based evidence.

## Data Availability

Not applicable.
